# Perceived personal, social and environmental barriers to weight maintenance among young women: A community survey

**DOI:** 10.1186/1479-5868-1-15

**Published:** 2004-10-05

**Authors:** Sari Andajani-Sutjahjo, Kylie Ball, Narelle Warren, Victoria Inglis, David Crawford

**Affiliations:** 1 Centre for Physical Activity and Nutrition Research, School of Exercise and Nutrition Sciences, Deakin University, Melbourne, AUSTRALIA

**Keywords:** barriers, physical activity, healthy eating, weight maintenance, overweight, obesity, young women

## Abstract

**Background:**

Young women are a group at high risk of weight gain. This study examined a range of perceived personal, social and environmental barriers to physical activity and healthy eating for weight maintenance among young women, and how these varied by socioeconomic status (SES), overweight status and domestic situation.

**Methods:**

In October-December 2001, a total of 445 women aged 18–32 years, selected randomly from the Australian electoral roll, completed a mailed self-report survey that included questions on 11 barriers to physical activity and 11 barriers to healthy eating (relating to personal, social and environmental factors). Height, weight and socio-demographic details were also obtained. Statistical analyses were conducted mid-2003.

**Results:**

The most common perceived barriers to physical activity and healthy eating encountered by young women were related to motivation, time and cost. Women with children were particularly likely to report a lack of social support as an important barrier to physical activity, and lack of social support and time as important barriers to healthy eating. Perceived barriers did not differ by SES or overweight status.

**Conclusions:**

Health promotion strategies aimed at preventing weight gain should take into account the specific perceived barriers to physical activity and healthy eating faced by women in this age group, particularly lack of motivation, lack of time, and cost. Strategies targeting perceived lack of time and lack of social support are particularly required for young women with children.

## Introduction

In many developed countries, overweight and obesity have reached epidemic proportions [1-8]. One group at particular risk of weight gain and the development of obesity is young women[2,9,10]. In the US, for example, one study that tracked weight in a large population sample over a 10-year period found that major weight gain (increased body mass index (BMI) > 5 kg/m^2^) was twice as common in women (5.3%) as in men (2.3%) [2]. A recent study of almost 9,000 women aged 18–23 years in Australia showed that 41% of the sample gained more than 5% of their BMI baseline over a four-year period (1996–2000) [9]. This risk of weight gain and the development of obesity places young women at increased risk of a range of chronic medical conditions and diseases, such as hypertension, type-2 diabetes, cardiovascular disease, and certain cancers [11].

In an effort to reverse the current global epidemic of overweight and obesity, strategies to promote increased physical activity and to encourage healthy eating have been promoted in many countries [12-15]. In Australia, for instance, individuals are encouraged to consume diets that are low in fat, high in fibre and rich in fruits and vegetables[13], and to participate in at least 30-minutes of moderate-intensity activities at least five days/week [12]. Despite such efforts, many young women do not meet the current physical activity recommendations [16] and their diets are less than optimal. For example, mean daily intakes of fruits and vegetables fall well below recommended levels [17] and 50% of young Australian women are consuming at least one takeaway meal per week, which is likely to be high in energy density [9]. Poor compliance with dietary and physical activity guidelines is not unique to Australia [18-20]. In addition, recent work we have conducted suggests that many young women do not consider the kinds of lifestyle changes that are being recommended as feasible for them in the context of their daily lives [21]. An understanding of the perceived barriers faced by young women in achieving healthy lifestyle changes is therefore important.

Most existing studies examining perceived barriers to physical activity and healthy eating have focused on the general population,[18,22-25]. with few specifically considering the perceived barriers experienced by those at particular risk of weight gain, such as young women. However, the perceived barriers faced by young women are likely to differ from those faced by other groups, such as by men or older women. For example, a study in the USA showed that women more frequently report 'tiredness' and 'time' as significant perceived barriers to healthy habits than do men, and that this may be partly attributable to their domestic situation [25]. In addition, young women are more likely than older women to experience particular life events (e.g. leaving family homes, starting work, entering a marital or de facto relationship, and becoming mothers) that may influence their physical activity and dietary habits [26,27].

As well as perceiving different barriers to those faced by other groups in the population, the perceived barriers to increasing physical activity and improving diet that young women face may vary according to their social and personal circumstances. For example, having children is likely to impact on a women's ability to adopt healthy habits [21,28,29]. In addition, persons of lower socioeconomic status (SES) may have poorer access to parks, walking or jogging trails, and gym equipment than those of higher SES [25]. Access to good quality, inexpensive healthy foods has also been reported to be more limited among persons of low SES; for instance, the cost of healthy foods has been reported to be greater for those living in deprived areas. [30,31]. A number of studies have suggested that a lack of knowledge is a greater barrier to eating a healthy diet among those of lower education level [22,23]. Being overweight can also be perceived as a significant barrier to physical activity [32]. However, whether or not these factors are perceived as barriers to physical activity and healthy eating among young women is unknown.

In order to develop appropriate and effective obesity prevention strategies for young women it is important to understand the barriers they perceive in attempting to control their weight. The aim of this study was to examine perceptions of a range of personal, social and environmental barriers to physical activity and healthy eating, specifically related to weight maintenance, among young women, and how these vary by domestic situation, SES and overweight status.

## Methods

### Participants

A total of 445 women provided data for this study. Initially, a sample of 1200 women aged 18–32 years was selected from the Australian Electoral Roll using a stratified random sampling procedure, with strata based on the number of eligible cases in each of the eight States/Territories of Australia. As voting is compulsory for Australian adults, the electoral roll provides a complete record of population data on Australian residents aged 18 years and over. Excluding those who had moved and left no forwarding address, the study achieved a response rate of 41% (462 women participated), which is comparable to response rates reported in similar postal surveys with this age group [33,34]. Data from 17 women who were pregnant were excluded.

The socio-demographic characteristics of the sample are reported in full elsewhere [21]. Briefly, 42% of the respondents were tertiary-educated. Half of the women were married and one in three had at least one child. One in three respondents was classified as overweight or obese. The socio-demographic profile of the sample was comparable to that of women of similar age (18–44 y) who participated in the most recent (2001) Australian National Health Survey [35].

### Procedures

A questionnaire was developed and pilot-tested with a convenience sample of 10 women in the same age group as participants. The questionnaire, a study description, an invitation to participate, a consent form and a reply-paid envelope for returns were mailed to the study sample of women in October 2001. Non-responders were sent a reminder postcard two weeks later and a second reminder with replacement questionnaire a further three weeks later.

### Measures

The participants completed the following questions.

#### Socio-demographic background

The socio-demographic questions included domestic situation (household composition) and education. Domestic situation was assessed by asking 'Who lives with you?' with response options: *No-one*, *I live alone*; *Partner*/*spouse*; *Own children*; *someone else's children*; *parents*; *brothers*/*sisters*; *Other adult relatives*; and *Other adults who are not family members*. This was subsequently re-categorized as living with parental family; living alone/share 'flatting'; living with partner (no children); or living with children (including those living with partner and child/ren, and single mothers). Education level (highest level of schooling: *still at school*, *primary school*, *some high school*, *completed high school*, *technical*/*trade school certificate*/*apprenticeship*, *or University*/*tertiary qualification*) was subsequently categorized as tertiary educated or not tertiary educated and used as an indicator of SES.

#### Body weight

Women were asked to self-report their height and weight and this information was used to calculate body mass index (BMI = weight (kg)/height (m^2^)). Self-reported height and weight have been shown to provide a reasonably valid measure of actual height and weight for the purpose of investigating relationships in epidemiological studies [36]. Women were categorised as overweight (BMI ≥ 25) or not overweight (BMI < 25) [11].

#### Perceived barriers to weight maintenance

Young women's perceptions of barriers to weight maintenance were assessed using 22 items. Participants were asked 'How important are the following as barriers to you keeping your weight at the level you want?' The complete list of barrier items is included in Tables 1 and 2. These items were based on a review of the literature investigating barriers to weight maintenance behaviours in other population groups [22-25]. There were two sets of perceived barriers assessed, those related to physical activity and those to healthy eating. For each set of questions, participants were asked about access to information; motivation; enjoyment; skills; partner support and children's support (where relevant); friends' support; access; cost; time due to job demands; and time due to family commitments as possible barriers. Response options for all barrier items were: *Not a barrier*; *A somewhat important barrier*; *A very important barrier*; *Not applicable*. For analyses, responses *Not applicable *and *Not a barrier *were combined.

**Table 1:** Perceived barriers to physical activity (N = 445)

**Barriers to physical activity**	Factor loadings	Not a barrier (%)	A somewhat important barrier (%)	A very important barrier (%)
**Factor 1: Personal barriers to physical activity **(Eigenvalue = 4.21, 38% variance, Cronbach's alpha = 0.76)				
Do not have the motivation to do physical activity, exercise or sport	0.58	26	34	40
Not enjoying physical activity, exercise or sport	0.80	57	25	18
Do not have the skills to do physical activity, exercise or sport	0.70	81	14	5
**Factor 2: Social support barriers to physical activity **(Eigenvalue = 1.13, 10% variance, Cronbach's alpha = 0.68)				
No partner's support to be physically active	0.80	78	13	9
No children's support to be physically active	0.82	94	4	2
No friends' support to be physically active	0.57	84	11	5
**Factor 3: Environmental barriers to physical activity **(Eigenvalue = 1.22, 11% variance, Cronbach's alpha = 0.71)				
Do not have enough information about how to increase physical activity	0.75	83	12	5
Not having access to places to do physical activity, exercise or sport	0.57	66	23	11
Not being able to find physical activity facilities that are inexpensive	0.70	49	29	22
Not having the time to be physically active because of job	0.76	42	29	29
Not having the time to be physically active because of family commitments	0.68	63	22	15

**Table 2:** Perceived barriers to healthy eating (N = 445)

**Barriers to healthy eating**	Factor loadings	Not a barrier (%)	A somewhat important barrier (%)	A very important barrier (%)
**Factor 4: Personal and environmental barriers to healthy eating **(Eigenvalue = 4.61, 42% variance, Cronbach's alpha = 0.83)				
Do not have enough information about a healthy diet	0.70	72	17	11
Do not have the motivation to eat a healthy diet	0.70	34	41	25
Do not enjoy eating healthy foods	0.80	64	26	10
Do not have the skills to plan, shop for, prepare or cook healthy foods	0.70	73	19	8
Do not have access to healthy foods	0.65	80	16	4
Not able to buy healthy foods that are inexpensive	0.60	60	27	13
**Factor 5: Social and environmental barriers to healthy eating **(Eigenvalue = 1.23, 11% variance, Cronbach's alpha = 0.72)				
No partner's support to eat a healthy diet	0.76	79	13	8
No children's support to eat a healthy diet	0.80	97	2	1
No friends' support to eat a healthy diet	0.57	83	12	5
Not having time to prepare or eat healthy foods because of job	0.47	57	23	20
Not having time to prepare or eat healthy foods because of family commitment	0.55	77	15	8

#### Most important perceived barriers

In order to ascertain women's perceptions of the single most important barrier to physical activity and healthy eating (which may not have been included in the list of barriers developed by the researchers), participants were asked the following two open-ended questions: 'What is the one thing that makes it hardest for you to be physically active?' and 'What is the one thing that makes it hardest for you to eat a healthy diet?'

### Statistical Analyses

Analyses were conducted mid-2003, using SPSS version 11.0.0 statistical software. [37]. Initially, descriptive analyses were performed to describe the proportion of women rating each of the items as not a barrier, a somewhat important barrier or a very important barrier. Content analyses of the open-ended questions were undertaken to identify main recurring themes.

Two separate exploratory factor analyses using SPSS FACTOR were performed with the 11 barriers to physical activity and the 11 barriers to healthy eating, to identify underlying patterns of relationships among individual items, and to reduce and simplify the items in order to facilitate subsequent analyses. Principal components analysis with varimax rotation (since factors were not correlated) was used. For any cross-loading items (i.e. items that had loadings of greater than 0.4 on more than one factor), only the higher loading was taken into account when calculating final factor scores. Inter-item reliability for each factor was assessed by Cronbach's α coefficients. Kaiser's measure of sampling adequacy was used to confirm the appropriateness of factor analysis [38]. Standardized factor scores were computed for each factor, with a large positive score representing more important barriers and a large negative score, less important barriers. Analysis of variance or t-tests were performed separately for each of the standardized factor scores to investigate differences in perceived barriers to physical activity and healthy eating with regard to domestic situation, SES and overweight status.

## Results

### Perceived barriers to physical activity

Table 1 presents the proportions of women reporting each of the perceived barriers to physical activity. The main barriers reported by young women related to motivation, time and cost. Combining the response categories 'somewhat important' and 'very important', 74% of the sample reported lack of motivation – 'not having the motivation to do physical activity, exercise or sport', time (58%) – 'not having time to be physically active because of my job,' and cost (51%) – 'not being able to find physical activity facilities that are inexpensive' – as common barriers to physical activity. Lack of time due to work commitments (reported by 58%) was more commonly reported than lack of time due to family commitments (37%), perhaps due to the relatively small proportion (30%) of young women in this study with at least one child. Less common perceived barriers to physical activity included lack of information, skills, partners' and children's support, and friends' support.

### Perceived barriers to healthy eating

Table 2 presents perceived barriers to healthy eating. As with physical activity, lack of motivation (66%), lack of time due to job commitments (43%), and cost (inability to buy healthy foods that are inexpensive: 40%) were common perceived barriers. Less commonly reported barriers included lack of information, skills and friends', partners' and children's support, and access. As with physical activity, lack of time related to job demands (reported by 43%) was more common than lack of time due to family commitment (23%).

### The most important perceived barriers to physical activity and healthy eating

Consistent with women's responses to the closed-ended questions, the most important perceived barriers to physical activity reported in response to the open-ended questions were lack of time due to work, study or family commitments (78%), lack of motivation (37%) and childcare issues (25%). The most important perceived barriers to healthy eating related to taste (24%); lack of time (21%); lack of motivation (13%); and the perception that healthy foods are inconvenient or expensive (13%).

### Factor analysis of perceived barriers to weight maintenance

The factor analysis of the perceived barriers to physical activity revealed three interpretable factors (Table 1) with eigenvalues greater than one. These factors together explained 60% of the total variance. Two items – 'not having access to places to do physical activity, exercise or sport' and 'not having friends' support to be physically active' – cross-loaded on two factors and these items were included only on factors on which each item showed the largest loading. The Cronbach's α coefficients for the three factors ranged from 0.68 to 0.76, indicating moderate internal reliability. Provisional names were assigned for these three factors: 'personal barriers', 'social support barriers' and 'environmental barriers'. The items included as personal barriers to physical activity were related to motivation, enjoyment, and skill. Social support barriers encompassed lack of support from family and friends; and environmental barriers related to information, access, cost, and time.

The principal components analysis of the 11 barriers to healthy eating resulted in two distinct interpretable factors with eigenvalues greater than one (Table 2). The Cronbach's α coefficients for the two factors were 0.72 and 0.83, indicating moderate to good internal reliability. Together the two factors explained 53% of the total variance. Provisional names were assigned to these factors: 'personal and environmental barriers' and 'social and environmental barriers'. Personal and environmental barriers to healthy eating included motivation, enjoyment, skills, information, cost, and access. Social and environmental barriers were related to lack of support from family and friends and time constraints.

### Associations of domestic situation, education and overweight status with perceived barriers

Mean factor scores did not vary according to women's overweight status or SES. Mean factor scores did differ significantly by domestic situation for two factors: social support barriers to physical activity and social and environmental barriers to healthy eating (see Table 3). Compared with women living in other domestic situations, women with children had the lowest score on the social support for physical activity factor, suggesting that lack of support from partners, children and friends was a more important perceived barrier to physical activity for these women. This group also had the lowest score on social and environmental barriers to healthy eating factor, suggesting that lack of social support and insufficient time were more important perceived barriers to healthy eating among women with children than among other women. Conversely, young women who lived with their parents had the highest scores on these factors, indicating the relative lack of importance of social support for physical activity, and social and environmental barriers to healthy eating, for this group.

**Table 3:** Mean standardized factor scores on weight maintenance by domestic situation^a^

**Factor**	**Domestic situation**				
	
	Parents	Alone/ Share	Partner	Children	p
Personal barriers to physical activity	0.08	0.22	-0.09	-0.07	0.12
Social support for physical activity	-0.37	-0.14	-0.06	0.55	.000
Environmental barriers to physical activity	0.13	0.12	0.03	-0.18	0.11
Personal and environmental barriers to healthy eating	-0.04	0.23	0.02	-0.04	0.25
Social and environmental barriers to healthy eating	-0.30	-0.16	-0.06	0.49	.000

^a^. A large positive score represents more important barriers; a large negative score, less important barriers.

## Discussion

This study suggests that a lack of motivation, time constraints due to work, and cost issues are the key perceived barriers to maintaining weight faced by young women. Overall these findings support other research that has examined barriers to physical activity and healthy eating [18,22,25,39]. However, the present study is unique in providing an insight into the relative importance of a range of personal, social and environmental factors as perceived barriers to weight maintenance among young women, a high risk group for weight gain. Findings showed that young women tended to rate personal factors as key perceived barriers to physical activity and healthy eating, followed by environmental factors, with social factors rated as less important. While the environment is likely to be an important source of influence on obesity-related behaviours [40], these findings highlight that efforts to prevent obesity should not ignore the central role of cognitive factors. Given the striking similarities in the types of barriers reported to impede physical activity, and the perceived barriers to healthy eating, findings also suggest that there may be potential economies of scale in health promotion programs aimed at preventing weight gain among young women. For example, strategies aimed at boosting motivation for healthy behaviour may help to promote both increased physical activity and healthy eating simultaneously. While motivating young healthy women to adopt healthy eating and physical activity behaviors is likely to be challenging, recent intervention research suggests that motivationally-tailored interventions may be more successful that other approaches (e.g. based on social-cognitive theory) in promoting physical activity and healthy eating [41,42].

It is noteworthy that perceived barriers to weight maintenance did not vary by socio-economic status or overweight status in this sample of women. In contrast, previous research has shown that overweight men and women face a number of perceived physical activity barriers [32]. Similarly, given that diet varies by socio-economic status [43,44] we expected that women of lower socio-economic status would be more likely to experience barriers to eating a healthy diet. Previous studies also suggest that persons of low SES often live in areas where the cost of food is greater, and access to healthy foods is poorer [30,31]. The reasons for the difference between the present results and earlier findings are unclear. It may be, however, that in this sample of relatively young women, many were still acquiring their education, and hence any SES differences in perceived barriers to healthy behaviours were not yet established.

Compared to other young women, those living with children were the most likely to report lack of social support for physical activity, and lack of support and time for healthy eating, as key perceived barriers to maintaining their weight. Young women who lived with their parents were the least likely to perceive these to be barriers to weight maintenance. These findings are consistent with those of previous studies showing that getting married and having children are associated with decreased physical activity and greater weight gain [21,26]. Any weight gain prevention program targeting women with children should incorporate a focus on enlisting social support for both physical activity, and shopping for and preparing healthy foods.

In a previous study with the same sample, we reported that while the majority of the women were in a healthy weight range (51%) or overweight/obese (31%), 18% of the women were underweight [21]. It should be acknowledged that some women in this sample, particularly those who were underweight, may have been trying to gain weight. One limitation of the present study was that the questions assessing perceived barriers to weight maintenance did not distinguish women trying to keep their weight down, from those trying to keep their weight up, and interpretation of the questions on perceived barriers may have been slightly different between these groups. However, attempts to gain weight are relatively uncommon among young women [45], and hence this is likely to have affected only a small proportion of the sample. A second limitation of this study is that the barriers were not assessed objectively, but rather through self-reports (ie perceived barriers). Nonetheless, it is important to consider women's perceptions of factors hindering their efforts to engage in healthy behaviours, since objective barriers may be perceived differently by different women (e.g., poor access to a gym may be viewed as less of a barrier to physical activity among a woman who walks for exercise than one who prefers aerobics). Finally, although the study achieved a somewhat modest response rate, the sample was selected from a nationally representative sampling frame and the socio-demographic profile of women was comparable to that of similarly-aged women in the wider population [35].

## Conclusions

The findings of this study highlight the need for health promotion strategies that provide increased motivation, support and skills to enable young women to shop and prepare healthy, quick and inexpensive meals. Similarly, the findings suggest a need to promote more time-efficient physical activity alternatives. Additional strategies that recognize the perceived barriers to physical activity and healthy eating faced by young women with children are particularly required.

## Competing interests

The authors declare that they have no competing interests.

## Authors' contributions

SA conducted the literature review, final statistical analyses and early drafts of the results and conclusions sections. KB and DC conceived the study, design and measures, collected the data, coordinated the analyses and participated in the write-up of all sections. NW conducted preliminary analyses and drafting of early results. VI contributed to drafting the final manuscript.
